# Pteridine levels and head weights are correlated with age and colony task in the honey bee, *Apis mellifera*

**DOI:** 10.7717/peerj.2155

**Published:** 2016-06-30

**Authors:** Frank D. Rinkevich, Joseph W. Margotta, Jean M. Pittman, James A. Ottea, Kristen B. Healy

**Affiliations:** 1 Department of Entomology, Louisiana State University Agricultural Center, Baton Rouge, LA, United States; 2 Honey Bee Breeding, Genetics, and Physiology Laboratory, USDA-ARS, Baton Rouge, LA, United States

**Keywords:** Honey bee, Pteridines, Polyethism, Fluorescence

## Abstract

**Background.** The age of an insect strongly influences many aspects of behavior and reproduction. The interaction of age and behavior is epitomized in the temporal polyethism of honey bees in which young adult bees perform nurse and maintenance duties within the colony, while older bees forage for nectar and pollen. Task transition is dynamic and driven by colony needs. However, an abundance of precocious foragers or overage nurses may have detrimental effects on the colony. Additionally, honey bee age affects insecticide sensitivity. Therefore, determining the age of a set of individual honey bees would be an important measurement of colony health. Pteridines are purine-based pigment molecules found in many insect body parts. Pteridine levels correlate well with age, and wild caught insects may be accurately aged by measuring pteridine levels. The relationship between pteridines and age varies with a number of internal and external factors among many species. Thus far, no studies have investigated the relationship of pteridines with age in honey bees.

**Methods.** We established single-cohort colonies to obtain age-matched nurse and forager bees. Bees of known ages were also sampled from colonies with normal demographics. Nurses and foragers were collected every 3–5 days for up to 42 days. Heads were removed and weighed before pteridines were purified and analyzed using previously established fluorometric methods.

**Results.** Our analysis showed that pteridine levels significantly increased with age in a linear manner in both single cohort colonies and colonies with normal demography. Pteridine levels were higher in foragers than nurses of the same age in bees from single cohort colonies. Head weight significantly increased with age until approximately 28-days of age and then declined for both nurse and forager bees in single cohort colonies. A similar pattern of head weight in bees from colonies with normal demography was observed but head weight was highest in 8-day old nurse bees and there was no relationship of head weight with age of foragers.

**Discussion.** Although the relationship between pteridine levels and age was significant, variation in the data yielded a +4-day range in age estimation. This allows an unambiguous method to determine whether a bee may be a young nurse or old forager in colonies with altered demographics as in the case of single cohort colonies. Pteridine levels in bees do not correlate with age as well as in other insects. However, most studies used insects reared under tightly controlled laboratory conditions, while we used free-living bees. The dynamics of head weight change with age is likely to be due to growth and atrophy of the hypopharyngeal glands. Taken together, these methods represent a useful tool for assessing the age of an insect. Future studies utilizing these methods will provide a more holistic view of colony health.

## Introduction

Insect age directly affects behavior, vectorial capacity, reproductive output, as well as insecticide detoxification and susceptibility. Under normal circumstances, age largely dictates the progression of tasks a honey bee worker performs for the colony by way of a juvenile hormone regulatory mechanism (Huang & Robinson, 1996; Robinson, 1992). As adult bees age, they transition from nursing young larvae, cleaning brood cells and building wax comb, to storing pollen and nectar, defending the entrance, and ultimately foraging for nectar and pollen outside of the colony. This stereotyped progression of behaviors is highly plastic, and disturbances to colony demography, food availability, and seasonal changes may induce reversions between behavioral states (Robinson, 1992). The transition from nurse to forager accompanies an extensive physiological rearrangement and dramatic increases in metabolic rate (Harrison, 1986; Harrison & Fewell, 2002). From a toxicological perspective, honey bee age affects insecticide sensitivity (Rinkevich et al., 2015). Therefore, insecticide exposure may induce a demographic shift in the colony. Detecting changing demography in the colony provides a new way to interpret field-based studies on honey bee health and insecticide sensitivity.

Many methods exist for age determination in insects such as measuring ovarian follicle characteristics, counting cuticular bands, grading amount of fat body, and cuticular degradation (Hayes & Wall, 1999). However, most of these methods are tedious, do not allow processing many samples, based upon a single sex, and have limits of age determination. The use of pteridines to determine the age of an insect drastically reduces these limitations.

In 1891, pteridine pigments were first identified in butterfly wings (Hopkins, 1891). Pterdines consist of fused pyrimidine and pyrazine ring byproducts of purine metabolism, which are important for excretion, body coloration, and eye pigmentation (Wigglesworth, 1964; Zeigler & Harmsen, 1969). These pteridines accumulate with age, allowing accurate age determination by measuring pteridine concentration. The use of pteridines in aging has recently been evaluated in other insects, such as the ant, *Polyrhachis sexpinosa* (Robson & Crozier, 2009), and pink-spotted bollworm, *Pectinophora scutigera* (Noble & Walker, 1990). Using pteridines to determine insect age has been studied in a number of Dipteran species in which light intensity, sex, and temperature may affect the relationship of pteridines with age (Robson et al., 2006). However, an attempt to use pteridines to determine the age of the eusocial Hymenopteran ant, *Polyrachis sexpinosa*showed that only head weight, but not pteridine concentration were correlated with age (Robson & Crozier, 2009). To date, no published methods use pteridines to estimate bee age. Here, we report on using head pteridine concentration and head weight to determine the age of nurse and forager bees.

## Materials and Methods

### Honey bees

Frames of emerging brood were removed from colonies of Italian bees (*Apis mellifera ligustica*), cleared of all adult bees, and then placed in wooden enclosures (60 cm L × 15 cm W × 45 cm H) with wire mesh on the wide faces of the box. Frames were stored at 33 ± 1 °C with >70% RH in continuous darkness. The next morning, newly emerged bees were brushed off of the frames into a plastic tub (50 cm L × 40 cm W × 15 cm H) with a thin layer of petroleum jelly around the rim. Individual bees were marked with a dot of enamel paint (Testors, Vernon Hill, IL, USA) on the thoracic dorsum with a paint brush to identify bees of a known age.

To distinguish if pteridine accumulation was different between nurses and foragers, we set up Single-Cohort Colonies (SCCs) by housing more than 2,000 marked newly emerged bees in a five-deep frame nucleus box with one frame of honey and pollen, one frame of brood, and three frames of empty drawn out comb (Giray & Robinson, 1994). Each SCC was headed by an Italian queen (Wooten’s Golden Queens, Palo Cedro, CA, USA). The SCCs were stored at the environmental conditions mentioned above for 5 days, then placed in an apiary at the USDA-ARS Honey Bee Breeding, Genetics, and Physiology Research Unit in Baton Rouge, LA. Four SCCs were constructed from four independent batches of emerging brood from six source hives. A sample of three nurse bees and three forager bees were collected every 3–5 days until 42 days of age. Nurse bees were collected as they were actively nursing young larvae on brood comb. We were unable to collect nurse bees after 35 days. The entrances to the SCCs were blocked to collect returning foragers. A total of 390 bees were evaluated in the SCC study. Bees were frozen at −80 °C until pigment extraction later that same day.

For experiments in colonies with normal demography, marked bees were returned to the four respective colonies consisting of four deep boxes of brood and honey and approximately 30,000 worker bees. Nurse and forager bees were collected as above up to 38 days of age. A total of 125 bees were collected from colonies with normal demography.

### Pteridine extraction

Pteridines were extracted according to previously published methods (Robson & Crozier, 2009). Frozen bee heads were removed with a scalpel. Individual heads were weighed to the nearest mg and placed in a 1.5 mL tube on ice. A 0.5 mL volume of chloroform:methanol (2:1 v/v) was added to each tube. Heads were ground with a plastic pestle for 30 s. Samples were sonicated using a Q125 Sonicator (QSonica, Newtown, CT, USA) at 5 W for 15 s. Samples were placed on ice for >1 min, then sonicated for another 15 s. Tubes received 0.75 mL of 0.1 N NaOH (adjusted to pH 10 with 11.5 g/L glycine). All tubes were vortexed for 10 s then centrifuged for 5 min at 5,000× g at 4 °C. The 0.75 mL supernatant was saved and used for fluorescence determination. Pteridine fluorescence was measured with excitation at 355 nm (5 nm slit) and scanned in the emission spectra from 365 to 500 nm based on previously published methods (Robson & Crozier, 2009) on a Horiba FluoroMax fluorometer (Horiba Scientific, Kyoto, Japan) and the peak intensity was recorded. The ng of pteridines in the sample was calculated based on a standard curve of 6-biopterin (Mail & Lehane, 1988). The ng of pteridines was standardized by head weight (ng pteridines/mg head weight).

### Statistical analysis

All statistics were performed with JMP (SAS Institute, Cary, NC). All data were tested for normality. In order to determine the correlation between age and pteridine concentration, a linear regression analysis was performed. The slope of pteridine concentration for nurses and foragers was compared with a Student’s *t*-test. Total pteridine concentrations were compared between nurse and forager bees using Chi-Square analysis. The rate of increase was compared between nurse and forager bees using linear regression analysis.

## Results

### Single cohort colonies

**Figure 1:**
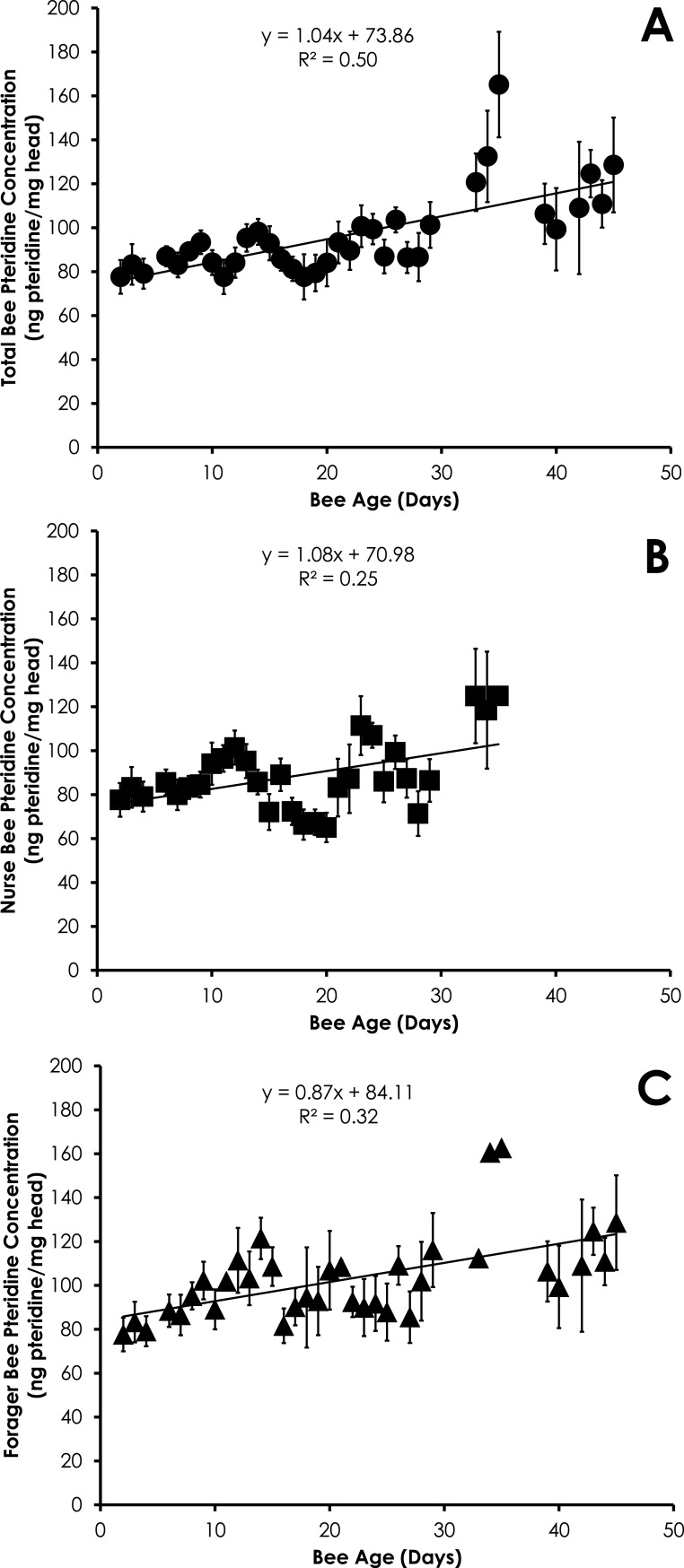
Linear relationship between bee age and pteridine concentration for all bees (A), nurse bees (B), and forager bees (C) from Single Cohort Colonies. Data are shown as average (±SEM).

Pteridine concentration significantly increased in a linear manner with age for all bees (*df* = 390, *F* = 19.96, *p* < 0.0001, *R*^2^ = 0.045, Fig. 1A), nurses (*df* = 212, *F* = 7.66, *p* = 0.0061, *R*^2^ = 0.035, Fig. 1B), and foragers (*df* = 178, *F* = 4.94, *p* = 0.0275, *R*^2^ = 0.027, Fig. 1C) from SCCs. Comparison of pteridine concentrations between nurses and foragers showed that foragers possessed significantly higher pteridine concentrations than nurses (*F* Ratio = 16.1, *p* ≪ 0.0001). The differences in pteridine concentrations between nurses and foragers became significantly wider with age (*R*^2^ = 0.37, *F* = 15.01, *p* = 0.001). However, there was no difference in the rate of increase in pteridine concentrations by comparison of the slopes for both nurses and foragers (*t* = 0.16, *df* = 71, *p* = 0.87), most likely due to the large error in the slopes.

**Figure 2:**
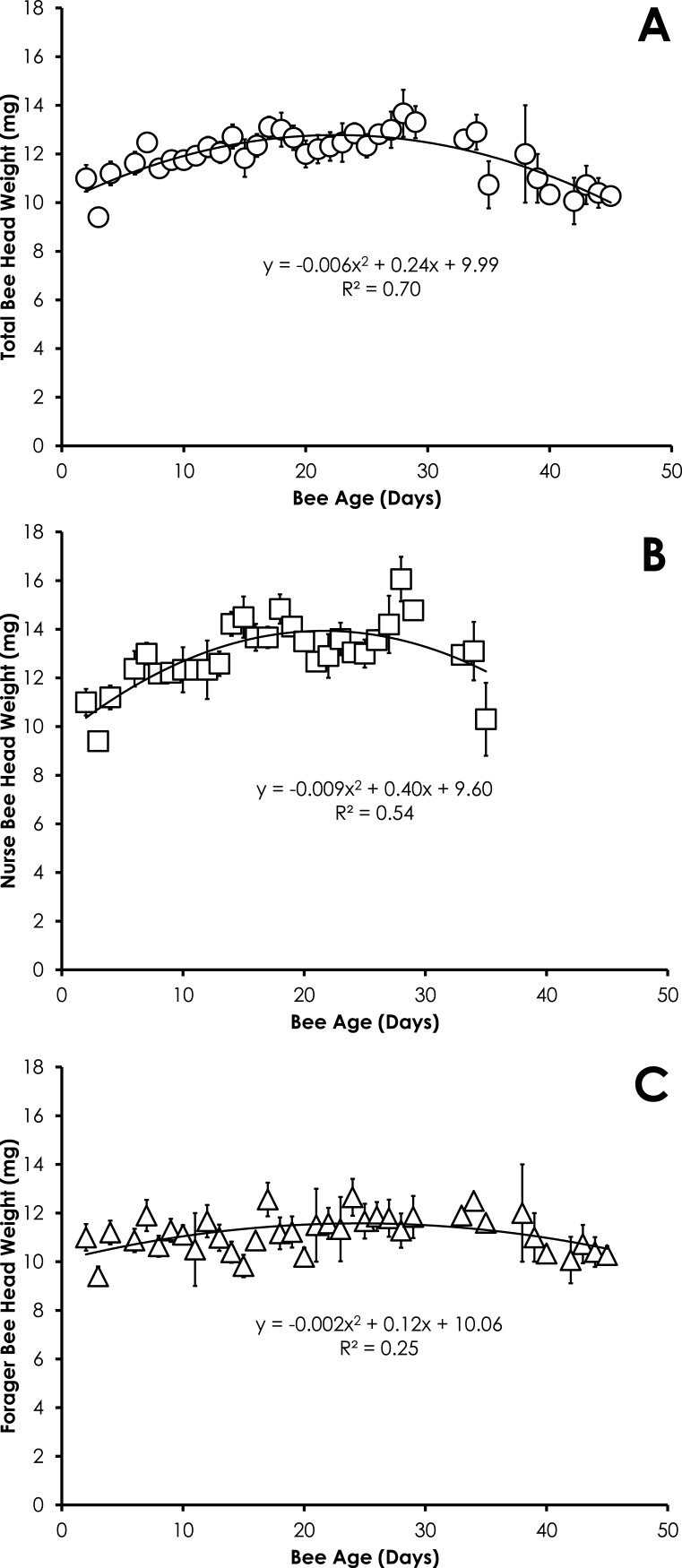
Quadratic relationship between bee age and head weight for all bees (A), nurse bees (B), and forager bees (C) from Single Cohort Colonies. Data are shown as average (±SEM).

Head weight for all bees in SCCs was significantly correlated with age, but the relationship was best fit with a quadratic equation. Head weight increased until 28 days of age, then declined through day 45 (Fig. 2A). The relationship between age and head weight was highly correlated (*R*^2^ = 0.70) and highly significant (*F* = 39.11; *p* < 0.001) for all bees (Fig. 2A). The relationship between age and head weight varied between nurses (*R*^2^ = 0.54) and foragers (*R*^2^ = 0.25), but remained highly significant in both cases (*F* = 16.18, *p* < 0.001; *F* = 5.71, *p* = 0.007, respectively). Nurses had significantly higher head weight than foragers on days 8–16 (Figs. 2B and 2C, *F* = 10.09, *p* ≪ 0.001).

**Figure 3:**
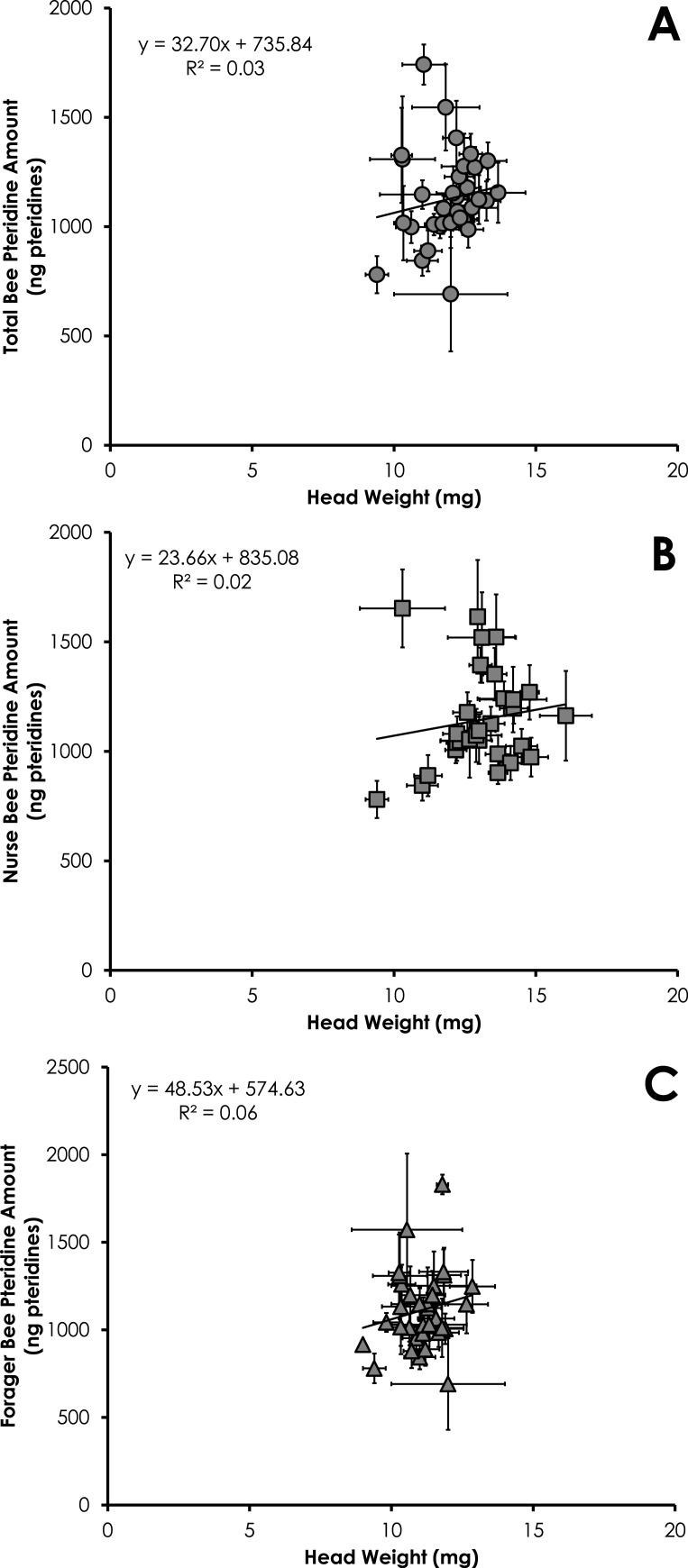
Linear relationship between head weight and pteridine amount for all bees (A), nurse bees (B), and forager bees (C) from Single Cohort Colonies. Data are shown as average (±SEM).

Total pteridine amount (ng pteridines not standardized by head weight) was significantly correlated with head weight from all bees from SCCs (*df* = 390, *F* = 14.10, *p* = 0.0002, *R*^2^ = 0.035, Fig. 3A). This relationship was significant for nurses (*df* = 212, *F* = 4.68, *p* = 0.03, *R*^2^ = 0.021, Fig. 3B) and foragers (*df* = 178, *F* = 11.09, *p* = 0.001, *R*^2^ = 0.06, Fig. 3C).

**Figure 4:**
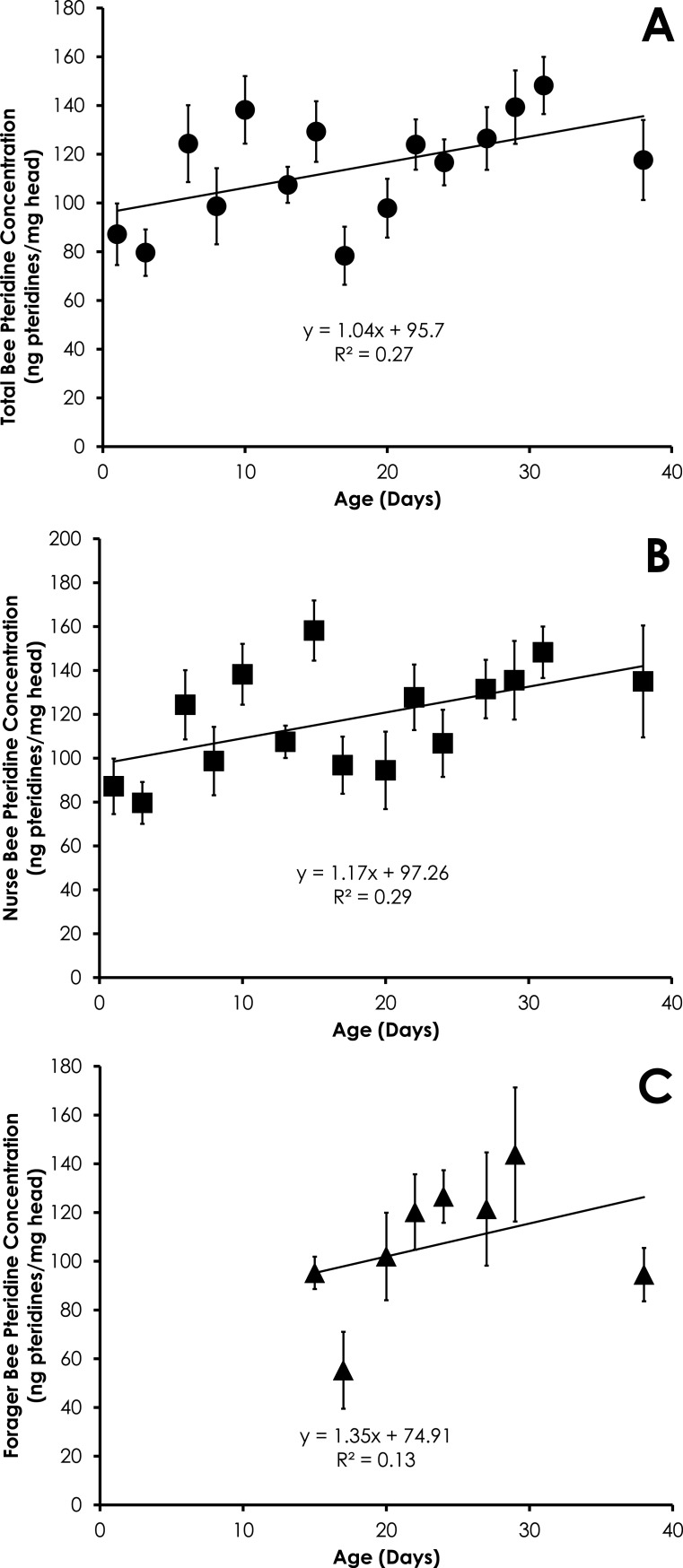
Linear relationship between bee age and pteridine concentration for all bees (A), nurse bees (B), and forager bees (C) from colonies with normal demography. Data are shown as average (±SEM).

### Colonies with normal demography

Pteridine concentrations significantly increased in a linear manner for all bees in colonies with normal demography (*F* = 4.92, *p* = 0.044, *R*^2^ = 0.27, Fig. 4A). Pteridine concentrations in nurse bees from colonies with normal demography significantly increased in a linear manner (*F* = 5.45, *p* = 0.036, *R*^2^ = 0.29, Fig. 4B). There was no significant relationship between age and pteridine concentrations in foragers from colonies with normal demography (*F* = 0.94, *p* = 0.36, *R*^2^ = 0.13, Fig. 4C). There was no difference in the slopes of the relationship between pteridines and age among nurses and foragers from colonies with normal demography (*t* = 0.11, *df* = 19, *p* = 0.91).

**Figure 5:**
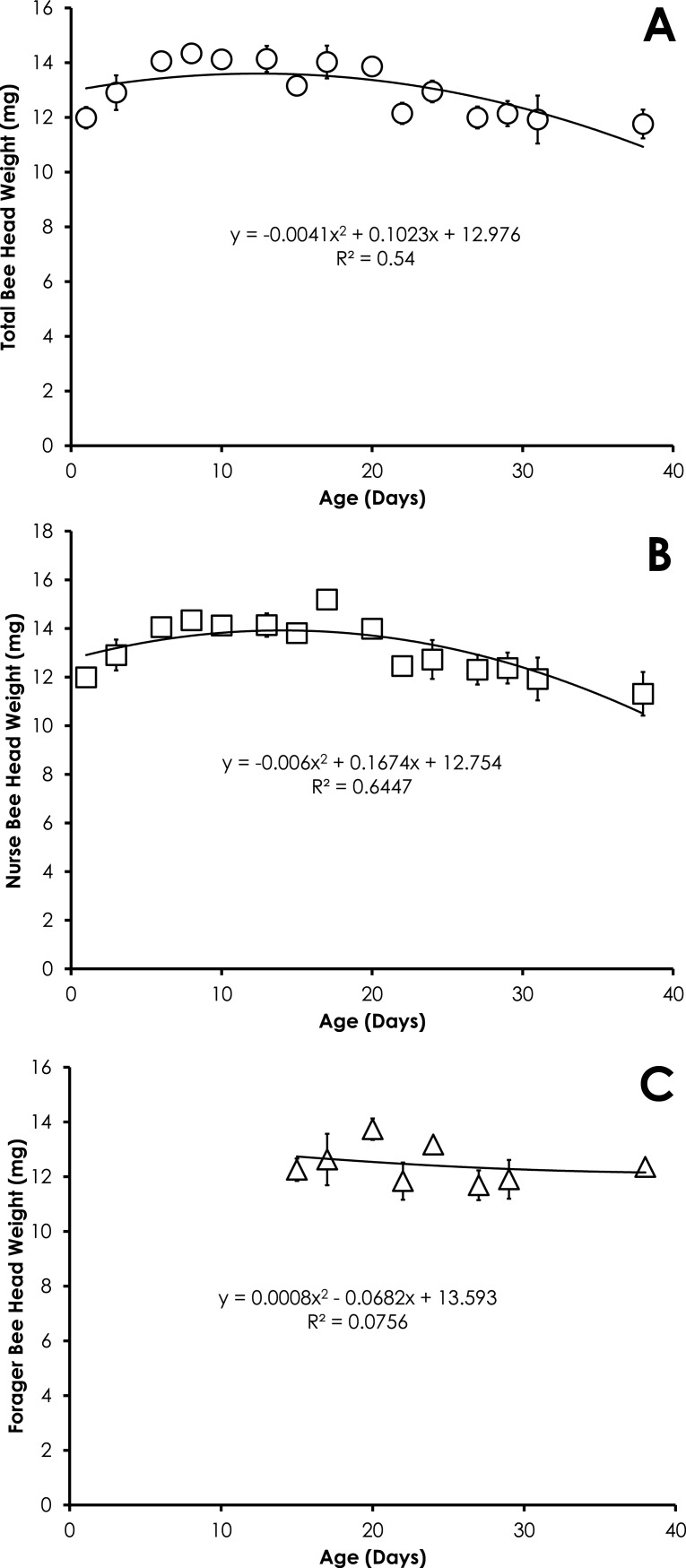
Quadratic relationship between bee age and head weight for all bees (A), nurse bees (B), and forager bees (C) from colonies with normal demography. Data are shown as average (±SEM).

Head weight for all bees in colonies with normal demography was significantly correlated with age that was best fit with a quadratic equation. Head weight increased until 8 days of age, then declined through day 38 (Fig. 5A). The relationship between age and head weight was highly correlated (*R*^2^ = 0.54) and highly significant (*F* = 7.04; *p* = 0.0095) for all bees from colonies with normal demography (Fig. 5A). A similar significant quadratic relationship was seen in nurses (*F* = 10.88, *p* = 0.002, *R*^2^ = 0.64, Fig. 5B). Head weight was not significantly correlated with age of foragers (*F* = 0.20, *p* = 0.82, *R*^2^ = 0.075, Fig. 5C).

**Figure 6:**
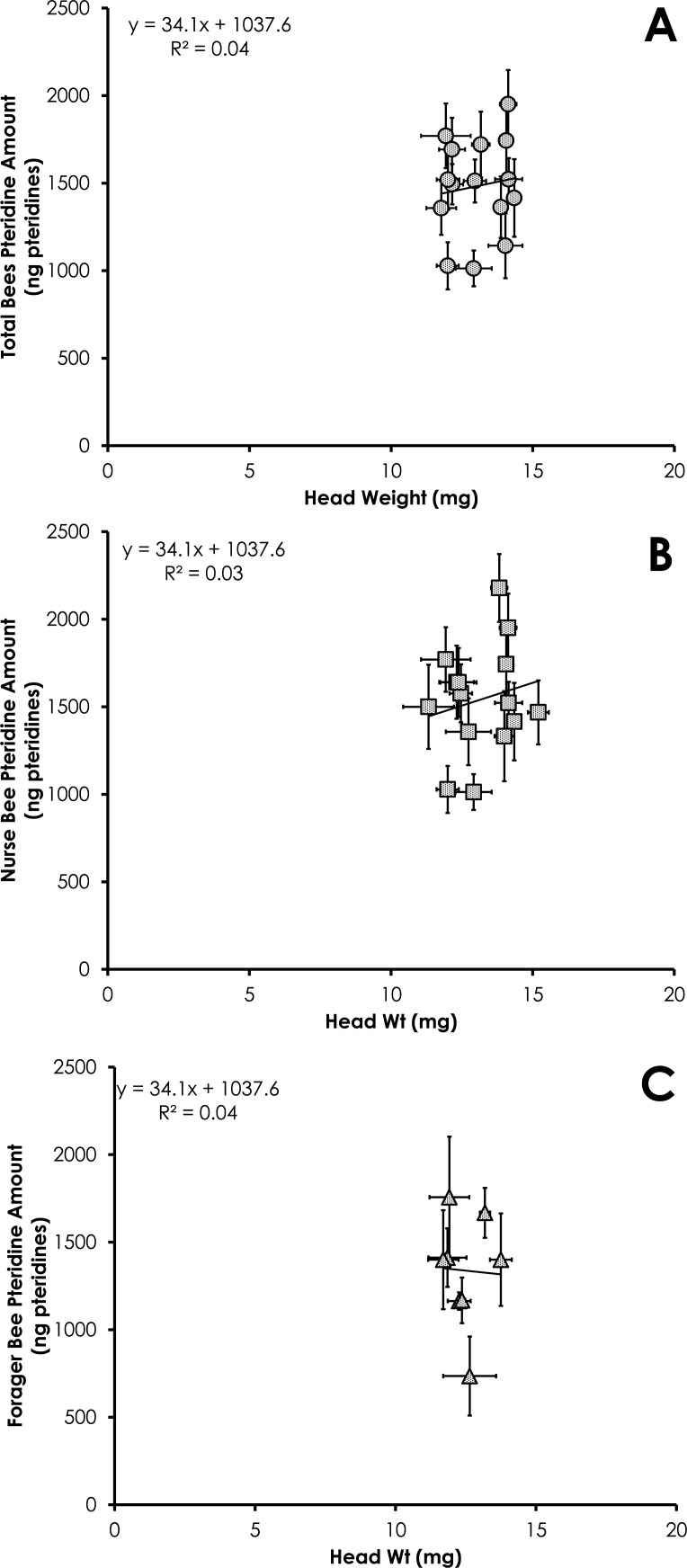
Linear relationship between head weight and pteridine amount for all bees (A), nurse bees (B), and forager bees (C) from colonies with normal demography. Data are shown as average (±SEM).

Total pteridine amount (ng pteridines not standardized by head weight) was significantly correlated with head weight from all bees from colonies with normal demography (*df* = 124, *F* = 4.92, *p* = 0.028, *R*^2^ = 0.038, Fig. 6A). This relationship was not significant for nurses (*df* = 86 , *F* = 2.37, *p* = 0.12, *R*^2^ = 0.027, Fig. 6B) or foragers (*df* = 37, *F* = 1.47, *p* = 0.23, *R*^2^ = 0.039, Fig. 6C).

## Discussion

Pteridine concentration significantly correlated with bee age, and our results suggest this method can be used to compare nurse and forager bees from colonies with altered demography. There was no difference in the pteridine concentrations between nurses and foragers in colonies with normal demography, but there was a significant difference between nurses and foragers in SCCs. Comparison of the type of colony used (SCC vs. Normal) showed a very significant effect (Standard Least Squares, Effect Leverage, *t* = 3.90, *df* = 82, *p* = 0.0002). We divided the forager results from the SCCs into two groups of bees: 14-days of age or less and 15-days of age or more based on the age when foragers appeared in colonies with normal demography at 15 days of age. Comparison of these groups showed a significant difference in pteridine concentrations between precocious foragers 14 days of age or less and normal aged foragers (Standard Least Squares, Effect Leverage, Least Squares Means Differences Student’s *t*, *t* = 1.97, *p* < 0.05). After a stress event perturbs the demography of a hive, precocious foraging likely initiates, and this method may easily detect precocious foraging and demographic shifts in a colony as simulated in SCCs.

In precocious foraging, younger honey bees, which typically nurse inside of the hive, begin foraging at an earlier age. This type of response usually results because of stressors such as pests, pathogens, poor nutrition, or environmental parameters (Perry et al., 2015). Given the recent importance of colony declines to domestic bee keepers, determining precocious foraging in the field would be a beneficial tool to better understand the impact of these stressors.

Sex of the insect, ambient light intensity and temperature may affect pteridine concentrations (Robson et al., 2006). Since all worker bees are female, as well as the low light conditions and relatively constant temperature within the colony (Seeley & Heinrich, 1981), these factors are largely controlled in this study. The exception is when foragers leave the colony. We found that foragers have significantly higher pteridine concentrations than nurses in SCCs. This may be the result of the much higher light intensity encountered by foragers outside of the colony as increased light intensity has been shown to increase pteridine accumulation in *Drosophila serrata* (Robson et al., 2006). However, the lack of such a difference in colonies with normal demography suggests a complex regulation of pteridine concentrations in honey bees.

In this study, we used 6-biopterin as our standard in order to calculate concentration of pteridines from fluorescence. Wu & Lehane (1999) conducted HPLC recordings of fluorescent compounds in mosquitoes of different ages. Although they identified several pteridine compounds, 6-biopterin varied the most with age. Noble & Walker (1990) also isolated 6-biopterin from pink bollworm moths at increasing ages. However, their results did not show a correlation with age. Although their use in age has not been evaluated in bees, biopterin has been isolated from bees (Rembold & Hanser, 1960; Haydak & Vivino, 1950; Lingens & Rembold, 1959). Therefore, we are confident that use of 6-biopterin as our standard was an appropriate technique to estimate pteridine concentration in bees. However, future studies to isolate and quantify specific pteridines in bees via HPLC analysis should be considered.

The relationship of head weight with age varies less than the relationship of pteridine concentrations with age by comparison of the *R*^2^. However the quadratic nature of the relationship between head weight and age yields two date outcomes. In order to overcome some of these potential conflicts, any value that produces an age of <0 can be ignored and the older date should be considered as the true age. The age as determined by head weight would be more accurate if other characteristics are considered such as amount of hair on the body, wing wear, body pigmentation, and the behavioral state of the bee when it was collected.

The pattern of the increase in head weight is likely due to the fluctuation in the size of the hypopharyngeal gland (Hrassnigg & Crailsheim, 1998). The hypopharyngeal gland grows in size upon emergence to allow nurses to produce brood food. The hypopharyngeal gland atrophies at the transition from nurse to other colony tasks (Johnson, 2010). The precocious foraging in our SCC set up showed that the behavioral state affects the head weight of bees (Figs. 2B and 2C), suggesting that age alone is not the only factor affecting head weight. Head weight decreases in older nurse bees. Despite the fact the older nurse bees (>21-days old) with lighter head weight (and presumably smaller hypopharyngeal glands) were actively nursing at the time of collection, it is likely these overage nurses are less effective at producing brood food with atrophied hypopharyngeal glands. Smaller hypopharyngeal glands have reduced rates of protein synthesis (Huang, Otis & Teal, 1989). While overaged nurses (>15 days-old) rear smaller adults with developed ovaries (Wegener, Lorenz & Bienefeld, 2009), they have been associated with rearing queen larvae (Jung-Hoffman, 1966).

Results of our study showed pteridine amount was significantly correlated with head weight (Figs. 3A–3C and 6A). Studies simply reporting fluorescence intensity values correlated with age fail to take into account the head weight variation of the insect. Therefore, determining the concentration of pigment in relation to head weight is critical to avoid biased pteridine measurements.

To date, this is the first study to evaluate the use of pteridine pigments in the aging of bees. While this study did not find a highly accurate correlation of pteridines with age that was comparable to studies in medically and forensically important insects, we do see the value in being able to evaluate the age structure of a colony, as well as the likelihood of precocious foraging. We also feel that future studies that compare various treatment and control groups in the field could utilize this type of analysis to compare overall age structures as an additional measurement of colony health.

##  Supplemental Information

10.7717/peerj.2155/supp-1 Supplemental Information 1 Pteridines Raw Data

## Additional Information and Declarations

### Data Availability

The following information was supplied regarding data availability: The raw data has been supplied as Data S1.

## References

[ref-1] Giray T, Robinson GE (1994). Effects of intracolony variability in behavioral development on plasticity of division of labor in honey bee colonies. Behavioral Ecology and Sociobiology.

[ref-2] Harrison JF (1986). Caste-specific changes in honeybee flight capacity. Physiological and Biochemical Zoology.

[ref-3] Harrison JF, Fewell JH (2002). Environmental and genetic influences on metabolic rate in the honey bee, *Apis mellifera*. Comparative Biochemistry and Physiology Part A: Molecular & Integrative Physiology.

[ref-4] Haydak MH, Vivino AE (1950). The changes in the thiamine, riboflavin, niacin and pantothenic acid content in the food of female honeybees during growth with a note on the vitamin K activity of royal jelly and beebread. Annals of the Entomological Society of America.

[ref-5] Hayes EJ, Wall R (1999). Age-grading adult insects: a review of techniques. Physiological Entomology.

[ref-6] Hopkins FG (1891). Pigments in yellow butterflies. Nature.

[ref-7] Hrassnigg N, Crailsheim K (1998). Adaptation of hypopharyngeal gland development to the brood status of honey bee (*Apis mellifera* L.) colonies. Journal of Insect Physiology.

[ref-8] Huang ZY, Otis GW, Teal PEA (1989). Nature of brood signal activating the protein synthesis of hypopharyngeal gland in honey bees, *Apis mellifera* (Apidae: Hymenoptera). Apidologie.

[ref-9] Huang ZY, Robinson GE (1996). Regulation of honey bee division of labor by colony age demography. Behavioral Ecology and Sociobiology.

[ref-10] Johnson BR (2010). Division of labor in honey bees: form, function, and proximate mechanisms. Behavioral Ecology and Sociobiology.

[ref-11] Jung-Hoffman I (1966). Die determination von Konigin und arbeiterin der honigbiene. Zeitschrift fur Bienenforschung.

[ref-12] Lingens F, Rembold UH (1959). Über den Weiselzellenfuttersaft der Honigbiene, III. Vitamingehalt von Königinnen- und Arbeiterinnefuttersaft. Hoppe-Seyler’s Zeitschrift fur Physiologische Chemie.

[ref-13] Mail TS, Lehane MJ (1988). Characterisation of pigments in the head capsule of the adult stable fly *Stomoxys calcitrans*. Entomologia Experimentalis et Applicata.

[ref-14] Noble RM, Walker PW (1990). Pteridine compounds in adults of the pink spotted bollworm, *Pectinophora scutigera*. Entomologia Experimentalis et Applicata.

[ref-15] Perry CJ, Sovik E, Myerscough MR, Barron AB (2015). Rapid behavioral maturation accelerates failure of stressed honey bee colonies. Proceedings of the National Academy of Sciences of United States of America.

[ref-16] Rembold UH, Hanser G (1960). About the queen cells jelly of the honey bee, VI. The metabolism of biopterin in the honeybee. Hoppe-Seyler’s Journal of Physiological Chemistry.

[ref-17] Rinkevich FD, Margotta JW, Pittman JM, Danka RG, Tarver MR, Ottea JA, Healy KB (2015). Genetics, synergists, and age affect insecticide sensitivity in the honey bee, *Apis mellifera*. PLoS ONE.

[ref-18] Robinson GE (1992). Regulation of division of labor in insect societies. Annual Review of Entomology.

[ref-19] Robson SKA, Crozier RH (2009). An evaluation of two biochemical methods of age determination in insects (pteridines and lipfuscin) using the ant *Polyrachis sexpinosa* Latrielle (Hymenoptera: Formicidae). Australian Journal of Entomology.

[ref-20] Robson SKA, Vickers M, Blows MW, Crozier RH (2006). Age determination in individual wild-caught *Drosophila serrata*using pteridine concentration. Journal of Experimental Biology.

[ref-21] Seeley TD, Heinrich B, Heinrich B (1981). Regulation of temperature in the nests of social insects. Insect thermoregulation.

[ref-22] Wegener J, Lorenz MW, Bienefeld K (2009). Physiological consequences of prolonged nursing in the honey bee. Insectes Sociaux.

[ref-23] Wigglesworth VB (1964). The life of insects.

[ref-24] Wu D, Lehane MJ (1999). Pteridine fluorescence for age determination of *Anopheles* mosquitoes. Medical and Veterinary Entomology.

[ref-25] Zeigler I, Harmsen R (1969). The biology of pteridines in insects. Advances in Insect Physiology.

